# The use of premature chromosome condensation to study in interphase cells the influence of environmental factors on human genetic material

**DOI:** 10.1100/tsw.2006.210

**Published:** 2006-09-25

**Authors:** Vasiliki I. Hatzi, Georgia I. Terzoudi, Christina Paraskevopoulou, Vasilios Makropoulos, Demetrios P. Matthopoulos, Gabriel E. Pantelias

**Affiliations:** ^1^ Institute of Nuclear Technology and Radiation Protection, National Center for Scientific Research NCSR “Demokritos”, Athens, Greece; ^2^ Hellenic Institute for Occupational Health and Safety, ELINYAE, Athens, Greece; ^3^ University of Ioannina Agrinio Campus, Department of Environmental and Natural Resources Management, Agrinio 30100, Greece

**Keywords:** premature chromosome condensation, cell fusion, calyculin-A, lymphocytes, DNA damage, genotoxicity, sister chromatid exchanges, chromosomal damage, chromosome aberrations, cell cycle delay, chemicals, ionizing radiation

## Abstract

Nowadays, there is a constantly increasing concern regarding the mutagenic and carcinogenic potential of a variety of harmful environmental factors to which humans are exposed in their natural and anthropogenic environment. These factors exert their hazardous potential in humans' personal (diet, smoking, pharmaceuticals, cosmetics) and occupational environment that constitute part of the anthropogenic environment. It is well known that genetic damage due to these factors has dramatic implications for human health. Since most of the environmental genotoxic factors induce arrest or delay in cell cycle progression, the conventional analysis of chromosomes at metaphase may underestimate their genotoxic potential. Premature Chromosome Condensation (PCC) induced either by means of cell fusion or specific chemicals, enables the microscopic visualization of interphase chromosomes whose morphology depends on the cell cycle stage, as well as the analysis of structural and numerical aberrations at the G1 and G2 phases of the cell cycle. The PCC has been successfully used in problems involving cell cycle analysis, diagnosis and prognosis of human leukaemia, assessment of interphase chromosome malformations resulting from exposure to radiation or chemicals, as well as elucidation of the mechanisms underlying the conversion of DNA damage into chromosomal damage. In this report, particular emphasis is given to the advantages of the PCC methodology used as an alternative to conventional metaphase analysis in answering questions in the fields of radiobiology, biological dosimetry, toxicogenetics, clinical cytogenetics and experimental therapeutics.

## INTRODUCTION

Nowadays, there is a constantly increasing concern regarding the mutagenic and carcinogenic potential of a variety of harmful environmental agents to which humans are exposed in their natural and anthropogenic environments. Harmful environmental agents, exerting their effect on living organisms from the beginning of life, developed the appropriate evolutionary pressure that resulted in their defense mechanisms. Human technological development as well as human lifestyle introduced new series of harmful agents that, either alone or in combination, affect humans and all other organisms. These new agents exert their hazardous potential in humans' personal (diet, smoking, pharmaceuticals, cosmetics) and occupational environments that constitute part of the anthropogenic environment. The most important consequences of human exposure to environmental hazards are DNA damage induced either by direct or indirect binding to DNA. Direct-acting chemicals like DNA alkylating agents (e.g., mitomycin-C), cross-linking agents (e.g., methyl methanesulfonate), and oxygen radicals (e.g., hydrogen peroxide), bind covalently to DNA[1]. On the other hand, epigenetic agents may induce DNA damage via other processes, like alteration of function of cellular proteins (e.g., etoposide, doxorubicin); denaturation or degradation of DNA (e.g., actinomycin D, streptonygrin); inhibition of deoxyribonucleotide synthesis (e.g., deoxyadenosine, aphidicolin), of DNA synthesis, and of DNA repair mechanisms (e.g., heavy metals, 5-fluorodeoxyuridine, hydroxyuria, 3-aminobenzamide); production of labile DNA by a chemical reaction; and/or incorporation of abnormal precursors (e.g., polycyclic hydrocarbons, intercalating agents, hydroxylamine)[2,3,4,5,6].

The exposure of cells to genotoxic factors may result in changes at the chromatid level, such as sister chromatid exchanges (SCEs), as well as in structural chromosomal alterations such as chromosomal aberrations[4,7]. Chromosomal aberrations and other forms of DNA damage are the cause of many human genetic diseases and there is substantial evidence that chromosomal damage is involved in cancer development in experimental animals and humans[4,8,9]. According to Savage et al. (1976)[10] structural chromosomal aberrations are of two types: (1) *chromosome type aberrations* that can be chromosomal breaks, chromosome gaps, and chromosome exchanges between one, two, or more chromosomes; and (2) *chromatid type aberrations* that can be chromatid breaks, gaps, and exchanges within a single chromatid of a chromosome[10,11]. Moreover, SCEs are a result of reciprocal exchange of DNA between the sister chromatids of a replicating chromosome, and there is evidence that SCE formation is correlated with homologous recombination events[12], even though it still remains unclear whether SCEs have a direct impact on human health.

Until now, the conventional cytogenetic analysis of chromosomal damage as a result of exposure to environmental chemicals is mainly based on the microscopic analysis of chromosomes in metaphase[9,13,14,15,16,17,18,19]. However, many environmental genotoxic and/or carcinogenic agents are known to trigger activation of checkpoints that delay transition from $G_1$ (pre-DNA synthetic phase) to S phase (DNA synthesis) and from $G_2$ phase (post-DNA synthetic) to mitosis (M) while DNA is repaired[20,21]. A cellular arrest in $G_1$/S and $G_2$/M phases seems to be a phenomenon that permits cells to repair damaged DNA prior to replication and mitosis, respectively[4,22,23,24]. Examples of genotoxic agents that induce a $G_2$-phase accumulation in human cell lines are some alkylating factors, ionizing radiation, radiomimetic chemicals, as well as some anticancer drugs[23,25,26,27]. Consequently, conventional analysis at the metaphase level may underestimate the clastogenic and mutagenic potential of various chemicals, and mutagenic and carcinogenic environmental factors. This triggers the need to develop more sophisticated *in vitro* cytogenetic techniques for the quantification and monitoring of DNA damage in interphase cells in particular after exposure to potentially genotoxic agents[28,29,30,31,32,33]. Such an alternative technique has been provided through the development of the premature chromosome condensation (PCC) assay which allows the visualization and analysis of chromosomal damage in interphase cells (Figs. 1–3).

In this report, the advantages of the PCC methodologies used as an alternative to conventional metaphase analysis or in combination with other cytogenetic techniques are reviewed. Furthermore, the use of PCC in overcoming problems that could not be solved with the conventional metaphase analysis is presented, and particular emphasis is given to the potential use of PCC to elucidate the mechanisms underlying conversion of DNA damage into chromosomal damage. Additional applications of the PCC methodologies in the fields of radiobiology, biological dosimetry, toxicogenetics, clinical cytogenetics, and experimental therapeutics are described, and future perspectives are discussed.

## PCC METHODOLOGIES

### PCC Induction Using Cell Fusion

Central for the PCC assay is the fusion of interphase "test" cells with mitotic "inducer" cells that can be mediated either by incubation with Sendai virus[34,35,36] or by treatment with fusing agents like polyethylene glycol[37,38,39,40,41]. In general, the results obtained with the two methods of fusion are considered equivalent[41]. The original PCC protocol required the use of the UV-inactivated Sendai virus as an agent to induce cell fusion[34,35,42]. However, the preparation of effective Sendai virus samples was technically demanding and required considerable expertise. Also, the fusion by means of the Sendai virus requires cells with membranes especially receptive to the virus particles. Lymphocytes and lymphoid cell lines, for instance, cannot be satisfactorily fused[43]. In addition, cytogenetic laboratories potentially interested in this method are not always willing to handle viruses.

As an alternative to the Sendai virus, the well-known fusogen polyethylene glycol (PEG) was applied to PCC-induction procedures[37,43,44,45,46]. However, due to various technical disadvantages reported in the literature, PEG has not been widely used for PCC induction. The conditions for cell fusion and PCC induction have to be adjusted according to the varying cell sensitivities to PEG. Concentrations, molecular weight, exposure time, solution for the PEG dilution, and removal time have to be monitored. This made the use of PEG for PCC induction difficult on a routine basis[47]. A simple and reproducible PEG procedure protocol for PCC induction using cell fusion of human lymphocytes with rodent (CHO) mitotic cells (Fig. 1) was first reported in 1983 by Pantelias and Maillie[40].

Using the PCC assay by means of cell fusion, interphase cells that are either cycling, noncycling, or arrested can be visualized and analyzed. In the hybrids formed by cell fusion, the mitotic factors present in the donor mitotic cell dissolve the nucleus membrane and condense chromatin of the interphase "test" cell. More specifically, cells that have undergone PCC assume a morphology that is characteristic of the position of the interphase cell in the cell cycle: single chromatid per chromosome in $G_1$ phase, double chromatids per chromosome in $G_2$, and pulverized chromosome regions in S phase[40] (Fig. 2).

### Chemically Induced PCC

Chemically induced PCCs can be obtained by the use of the chemical compounds such as calyculin-A. Calyculin-A was initially isolated from the marine sponge *Discodermia calyx*[48]. It is a potent cell-permeable inhibitor of protein serine/threonine phosphatases types 1 and type 2A. Inactivation of these phosphatases leads to PCC in all phases of the cell cycle ($G_1$, S, $G_2$, M) and enables the visualization of the chromatin configuration in interphase cells[31,32,40,42,49,50]. The morphology of chemically induced PCCs varies according to the phase of the cell cycle (Fig. 3) in the same way as PCCs induced by means of cell fusion with two exceptions. Induction of PCC in resting cells such as $G_0$ lymphocytes cannot be obtained using phosphatase inhibitors. Also, in chemically induced PCCs at $G_2$ phase, the centromeres are not clearly visible, thus making a $G_2$-phase cell easily distinguishable from a metaphase cell[32]. Apart from calyculin-A, other phosphatase inhibitors have been used for the chemical induction of PCCs, such as okadaic acid[51,52,53]. However, it has been mentioned that the efficiency of the induction of PCC by calyculin-A is greater than that by okadaic acid[54]. Okadaic acid and calyculin-A have been shown to induce PCC in various cell systems such as fibroblasts and phytohemagglutinin (PHA)-stimulated human lymphocytes[55,56,57,58].

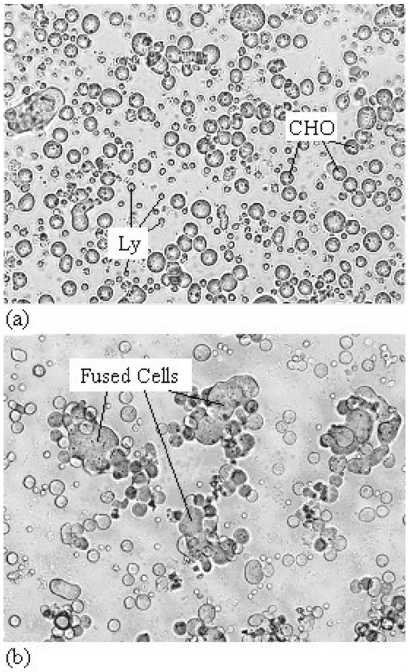

**FIGURE 1.** Fusion of lymphocytes with mitotic CHO cells in suspension as visualized under the microscope. (a) 10 min after fusion, (b) 60 min after fusion. CHO mitotic cells are larger than lymphocytes. Fused cells show a distinct appearance from either lymphocytes (Ly) or mitotic CHO cells. (Laboratory of Environmental Hygiene, Institute of Nuclear Technology and Radiation Protection, NCSR Demokritos, Microscope Nicon-Eclipse E400, magnification 200×)

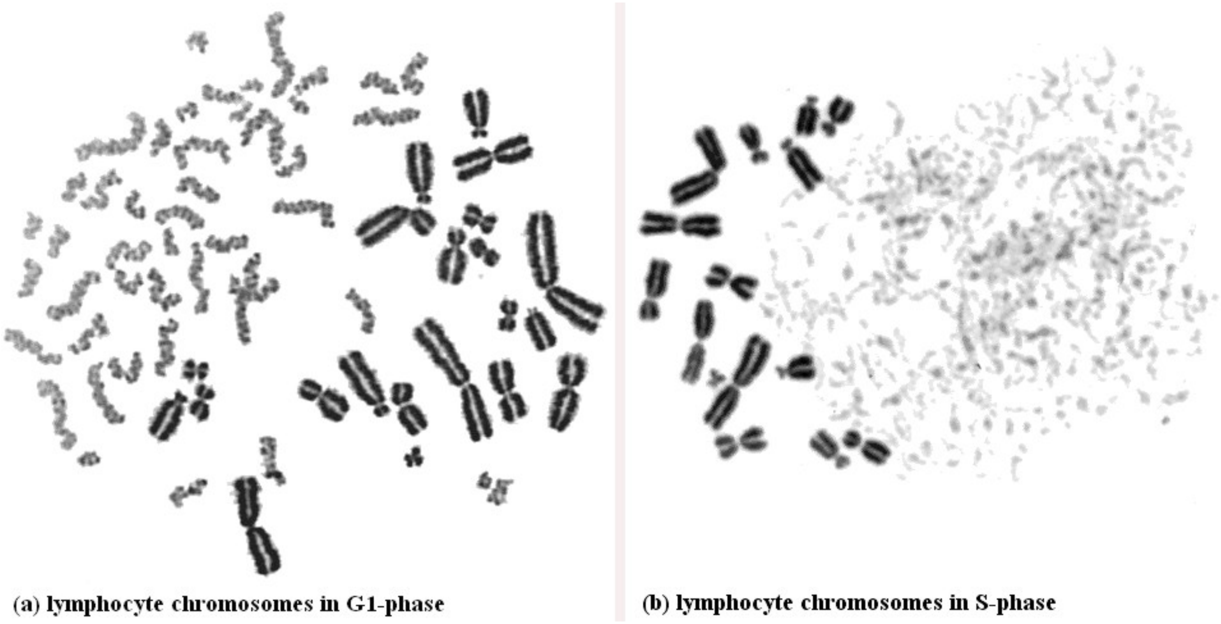

**FIGURE 2.** PCC mediated by cell fusion of human peripheral blood lymphocytes with rodent mitotic cells (CHO) in (a) G1 phase and (b) S phase. (Laboratory of Environmental Hygiene, Institute of Nuclear Technology and Radiation Protection, NCSR Demokritos, Microscope Nicon-Eclipse E400, magnification 1000×)

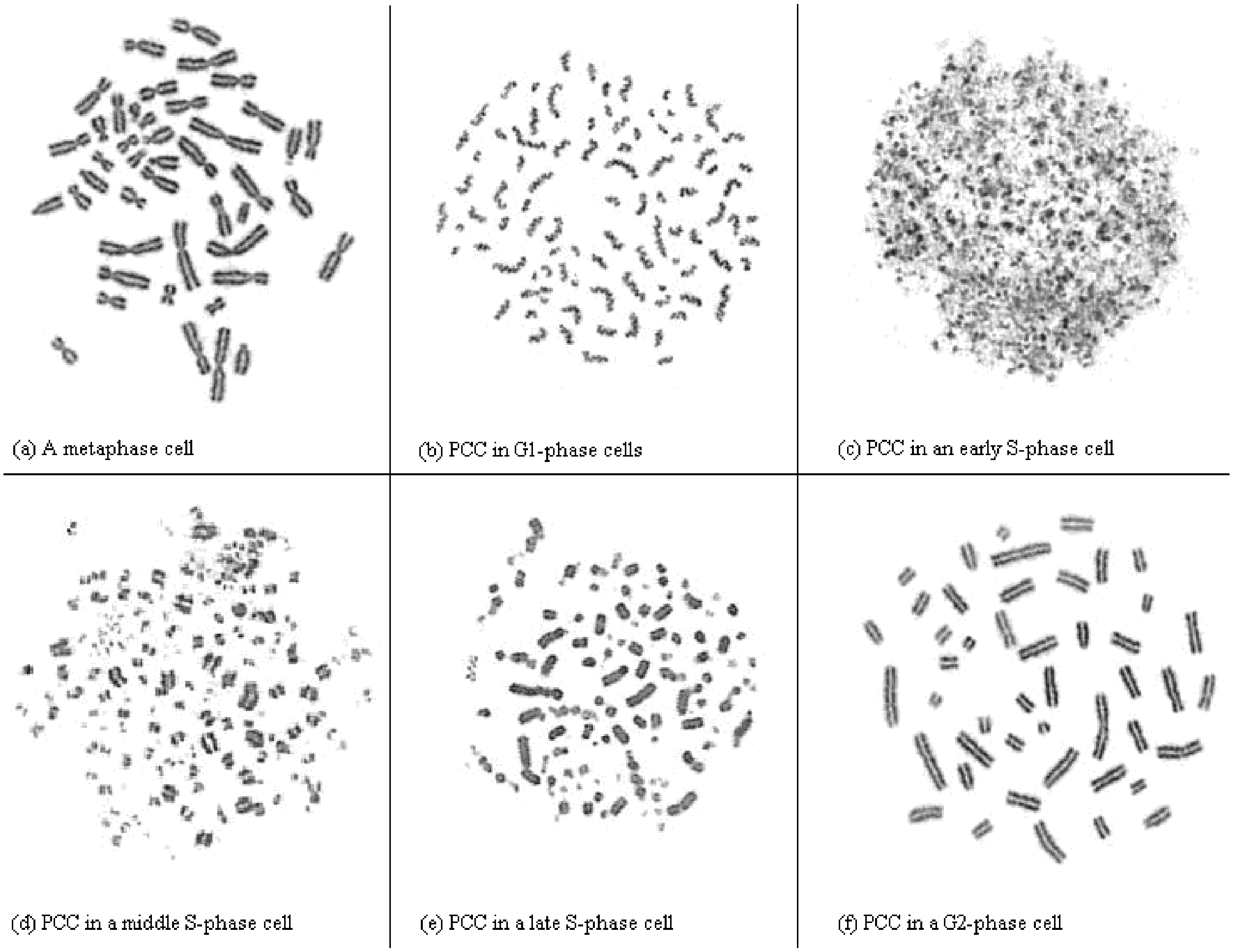

**FIGURE 3.** Microscopic images of human peripheral blood lymphocyte chromosomes. Lymphocytes were stimulated with PHA, cultured for 72 h, and treated with colcemid and 50n*M* calyculin-A for 30 min. (a) A metaphase cell, (b) a $G_1$-phase PCC, (c) an early S-phase PCC, (d) a middle S-phase PCC, (e) a late S-phase PCC, (f) a $G_2$-phase PCC. Note that in $G_2$-phase cells, the chromosome centromeres are not clearly visible. (Laboratory of Environmental Hygiene, Institute of Nuclear Technology and Radiation Protection, NCSR Demokritos, Microscope Nicon-Eclipse E400, magnification 1000×)

In the following paragraphs, the applications of the PCC methodologies and combinations of PCC with other cytogenetic techniques are reviewed.

## APPLICATIONS OF PCC METHODOLOGIES

### Biodosimetry and Biomonitoring of Exposure to Ionizing Radiation

Exposure of cells to ionizing radiation has been shown to cause a wide variety of phenomena, the most prominent of which are the induction of mutations, the induction of transformation, cell cycle arrest, and cell death. As a result, the study of phenomena elicited by radiation are of particular importance to human health and, therefore, elucidation of the underlying biochemical mechanisms and cytogenetic effects of ionizing radiation are a high priority in radiation biology research. In the field of biological dosimetry, numerous methods capable of detecting radiation-induced changes at the molecular, cytogenetic, and cellular level have been used in order to obtain absorbed dose estimates[50,59,60,61]. Scoring of dicentric chromosomes and centric rings at the first mitosis in cultured peripheral blood lymphocytes has been considered as one of the most reliable methods[50,60,62,63,64,65]. However, this procedure has several drawbacks. For example, some groups of individuals such as the elderly, patients with immunological diseases, or individuals that have been accidentally exposed to high doses of ionizing radiation, exhibit low mitotic indices (i.e., the percentage of cells undergoing mitosis) that do not allow the analysis of a sufficient number of cells in metaphase[62]. Moreover, it is well known that when cells are irradiated, most of them arrest in the $G_2$ phase because of DNA damage and these cells do not enter mitosis. As a result, it is very difficult to obtain a satisfactory number of mitotic chromosomes for analysis. In addition, only those cells reaching metaphase are collected and, thus, the scoring population might not be representative of the exposed population[66].

For these reasons, the PCC methodology was proposed as an alternative biodosimetric tool by Pantelias and Maillie in 1984[50] and since then it has been extensively used to assess and evaluate the induction and repair of chromosome damage after *in vivo* or *in vitro* exposure of human cells to ionizing radiation[25,67] A major benefit of the PCC assay, compared to the conventional analysis in metaphase, is that it does not require cells to divide for evaluation of cytogenetic damage. Due to this, PCC has been applied successfully in direct observation of radiation-induced cytogenetic damage in nonstimulated, interphase human lymphocytes[68] or CHO cells[69]. This application is of particular importance since it allows the visualization and scoring of radiation-induced chromosome damage in $G_1$ and $G_2$ cells immediately after irradiation (Fig. 4)[34,35,40,70,71]. Furthermore, induction of PCC allows the direct measurement of the rejoining kinetics of interphase chromosome breaks, as well as of the formation kinetics of ring and dicentric chromosomes due to ionizing radiation exposure[72,73,74].

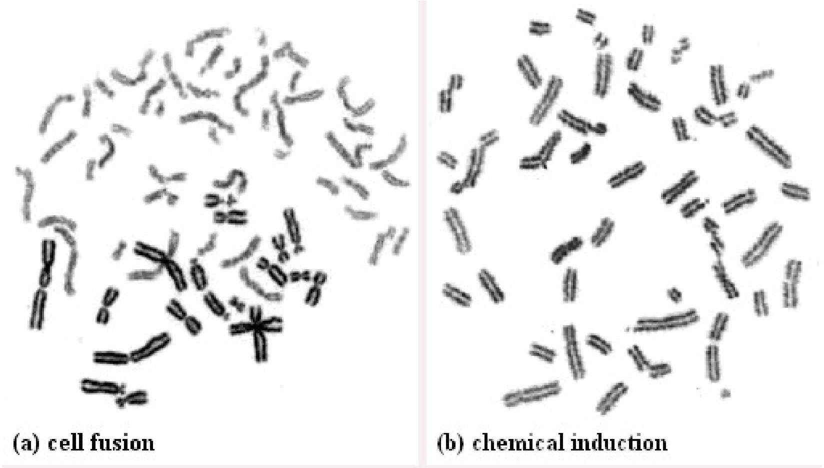

**FIGURE 4.** Chromatid breaks in cultured lymphocytes after $G_2$-phase irradiation as visualized directly in $G_2$ phase using PCC by means of (a) cell fusion and (b) chemical induction using calyculin-A. (Laboratory of Environmental Hygiene, Institute of Nuclear Technology and Radiation Protection, NCSR Demokritos, Microscope Nicon-Eclipse E400, magnification 1000×)

The need for in-depth analysis of genomic changes as a result of cellular exposure to ionizing radiation and genotoxic agents has triggered scientists to combine the PCC methodologies with other cytogenetic and molecular biology techniques. Numerous studies have coupled PCC methodologies in resting peripheral blood lymphocytes with fluorescent *in situ* hybridization (FISH) and chromosome painting for the analysis of numerical and structural chromosomal aberrations that are not visible using microscopic analysis with conventional staining procedures after exposure to genotoxic agents[52,75,76,77,78,79]. In radiobiology, the combination of chemically induced PCC with FISH has also been used to analyze the biological effectiveness of low and high LET (linear energy transfer)

radiation in the induction of chromosome damage in metaphase and interphase human lymphocytes[80]. The results demonstrated that chromosomal damage increased with LET in both interphase and metaphase cells, and that the number of chromosomal aberrations observed in interphase was threefold higher than in metaphase. Other scientific groups have combined chemically induced PCC in irradiated lymphocytes with pan-centromeric FISH in order to examine whether X-rays induce SCEs in ring chromosomes[51]. Gotoh and Asakawa[66] used okadaic acid–induced PCC in combination with chromosome painting to detect and evaluate chromosomal aberrations induced by high doses (>40 Gy) of γ-irradiation. Chromosomal aberrations in irradiated cells are detected by *in situ* hybridization using a centromere-specific probe followed by immunogold-silver painting. This simple method has allowed the detection of highly damaged chromosomes, to provide new information on the aberrations produced by high doses and to extend dose-response curves over a wider range[66]. PCC has also been used with autoradiography for the determination of damage induced by ionizing radiation of interphase cells in terms of chromosomal aberrations. According to this method, CHO cells in metaphase are exposed to various doses of X-rays up to 5 Gy, allowed to divide, and the results are obtained using autoradiographic analysis of chromosomes with [$^3$H]thymidine[37,39].

Several studies have proved that when the chromosomal analysis in interphase using PCC is combined with conventional staining cytogenetic techniques (G banding, Q banding) valuable information may arise[81,82]. When the PCC assay is combined with centromeric region staining (C-banding procedure), it enables the scoring of dicentric chromosomes, centric rings, and acentric fragments that are not visible in $G_1$-phase cells (Fig. 5). Measurement of dicentric chromosomes and centric rings in PCCs of human lymphocytes can be a powerful technique, not only in biodosimetry studies after *in vivo* exposure to ionizing radiation, but also genotoxicity studies for testing exposure to chemical agents[60].

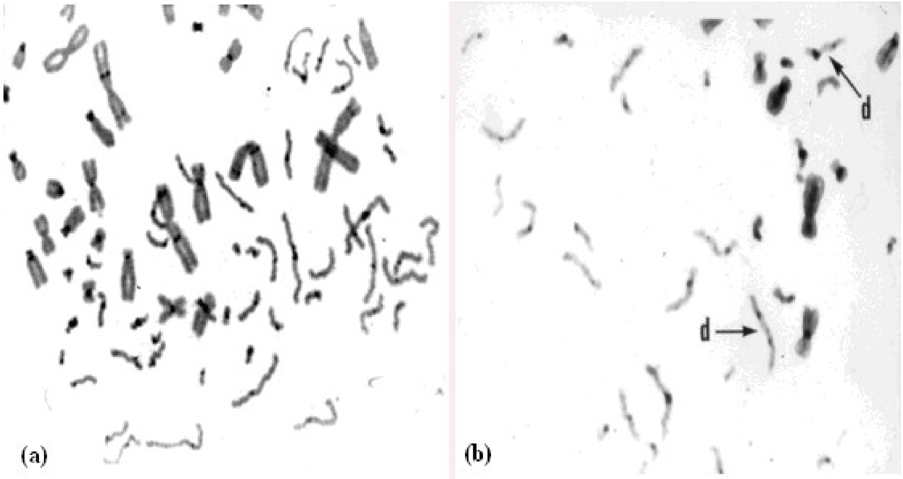

**FIGURE 5.** C-banded PCCs from (a) unirradiated and (b) irradiated lymphocytes (4 Gy of X-rays) showing dicentric chromosomes (see arrows).

## Mechanisms Underlying Conversion of DNA Damage into Chromosomal Damage

Visualizing cells with conventional analysis at metaphase can give information concerning only the residual damage after exposure to genotoxic environmental factors. The analysis of such interactions is based, therefore, only on those cells that proceed to mitosis. Therefore, it is difficult to elucidate the mechanism underlying the conversion of DNA damage into chromosomal damage. With the PCC methodologies, it is possible to gain valuable information not only to understand the biochemical mechanisms that affect the conversion of DNA damage into chromosomal damage, but also to determine possibly chromosomal radiosensitivity in $G_2$ phase, as well as variability in radiosensitivity at various stages of the cell cycle. The onset and the efficiency of chromatin condensation-decondensation are important determinants of these processes. Data obtained so far demonstrate the important role of cdk1/cyclin-B complex and of the $G_2$ checkpoint control mechanism in affecting chromatin conformation changes and conversion of DNA damage into chromosomal damage. Using the PCC method, it was realized specifically that changes in chromatin conformation soon after irradiation, presumably as a result of histone-H1 phosphorylation-dephosphorylation, strongly affected the conversion of DNA lesions into visual PCC fragments. The cdk1/cyclin-B complex was originally defined as the mitosis promoting factor (MPF), identified in mitotic frog eggs as a factor capable of inducing mitosis in $G_2$-phase cells. Regulation of cdk1/cyclin-B complexes at multiple levels ensures the tight regulation of the timing of mitotic entry[83].

In early reports, $G_0$ human lymphocytes were irradiated and analyzed at various times after fusion with mitotic CHO cells, i.e., as chromatin condensation proceeded. The yield of fragments observed was directly related to the amount of chromosome condensation allowed to take place after irradiation and inversely related to the extent of chromosome condensation at the time of irradiation. From these experiments, it was concluded that changes in chromosome conformation interfered with repair processes of DNA damage. In contrast, resting chromosomes (as $G_0$ lymphocytes irradiated before fusion) showed efficient repair of chromosomal damage. These results supported the hypothesis that DNA damage is converted into cytogenetic lesions and becomes observable when chromatin conformation changes occur during the cell cycle[39]. Furthermore, radiation-induced chromatid breaks in $G_0$ cells were analyzed by means of PCC using mitotic inducer cells with different cdk1/cyclin-B activities. The results showed that the higher the activity of mitotic PCC inducer, the higher the yield of chromatid breaks scored[84]. Also, the cdk1/cyclin-B activity during the $G_2$ to M transition has been shown to affect the conversion of DNA damage into PCC fragments[39,85], and it was suggested that variability in the cdk1/cyclin-B complex activity plays an important role in $G_2$-chromosomal radiosensitivity during the $G_2$/M transition checkpoint. Terzoudi et al. reported that the higher the cdk1/cyclin-B activity level of the cell line tested for $G_2$-chromosomal radiosensitivity, the higher the yield of chromatid breaks scored at metaphase after a certain dose in $G_2$ phase[86].

$G_2$ checkpoint facilitates repair of chromosomal damage, and the hypothesis that $G_2$-checkpoint defects during the $G_2$- to M-phase transition can also affect $G_2$-chromosomal sensitivity. This was tested using caffeine to abolish $G_2$ checkpoint by inhibiting ATM protein (Ataxia Telangiectasia mutated protein)[87]. The results in these studies have shown that *in vitro* treatment of lymphocytes obtained from healthy individuals or heterozygotes with caffeine, increased $G_2$-chromosomal radiosensitivity to the level of AT cells in agreement with earlier studies.

Concerning the variability of radiosensitivity to ionizing radiation at various stages of the cell cycle, it is already known that middle to S phase and $G_1$ phase are resistant stages, while mitosis, $G_1$/S, and $G_2$/M transition are very sensitive to radiation[85,88]. Moreover, ionizing radiation causes perturbation in the cell cycle progression, particularly lengthening of the $G_1$ and $G_2$ phases. Several possible mechanisms have been suggested to explain this variation in sensitivity and the perturbation in cell cycle progression following irradiation. It is mainly considered that induction and repair processes of DNA double strand breaks underlie the fluctuation in radiosensitivity observed at different stages of the cell cycle. An alternative hypothesis has been proposed, however, by Terzoudi and Pantelias[74,85] to explain such variability in radiosensitivity during progression in cell cycle. Chromosomal aberrations are not necessarily the result of irreparable double strand breaks, but rather a result of changes in chromatin conformation that works as an active conversion process of DNA damage into chromosomal damage. Activation of this mechanism is genetically determined, cell cycle–stage dependent, and may therefore explain the fluctuations in radiosensitivity at different stages of the cell cycle.

## Genotoxicity of Exposure to Chemical Agents

It is well known that increased rates of cell proliferation can escalate the risk of malignancy following exposures to chemical agents[89]. Since the knowledge about the mechanisms and effects of various genotoxic agents is critical to human health, the analysis of chromosomes in interphase cells seems to be extremely important in genotoxicity studies. The PCC method can be used to evaluate the effects on the fragility of chromatin structure, on the formation of chromosomal aberrations and SCEs (Fig. 6**)** as well as on cell cycle progression, as a result of exposure to chemicals agents[31].

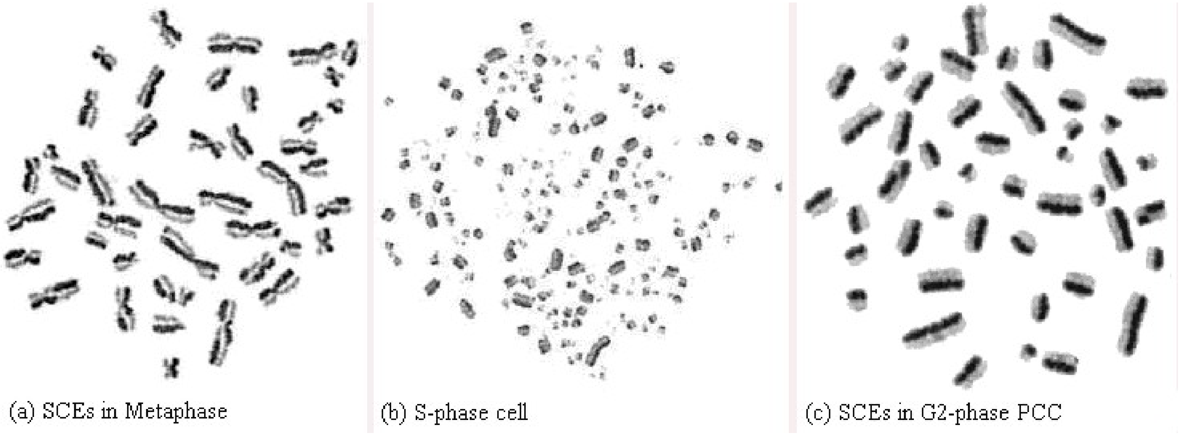

**FIGURE 6.** SCEs as visualized in (a) metaphase, (b) S phase and (c) $G_2$ PCCs of peripheral blood lymphocytes treated with 20 μ*M* of 5-bromodeoxyuridine for 48 h and 50 n*M* calyculin-A for 1 h. (Laboratory of Environmental Hygiene, Institute of Nuclear Technology and Radiation Protection, NCSR Demokritos, Microscope Nicon-Eclipse E400, magnification 1000×)

Numerous chemicals and genotoxic factors, like alkylating agents (e.g., mitomycin), radiomimetic chemicals (e.g., bleomycin), as well as chemicals found in the occupational environment (e.g., hydroquinone, paraquat, atrazine), have been shown to induce cell cycle delay in order to permit cells to repair the DNA damage[4,22,23,24,31,32,90]. Due to the induction of cell cycle arrest following exposure to these agents, a major problem that arises in genotoxicity studies is that although exposures to higher concentrations of an examined chemical could provide clear evidence for its genotoxicity, conventional analysis at metaphase cells cannot be used because affected cells are delayed in $G_2$ phase and do not proceed to mitosis. Moreover, even though the use of higher chemical doses could clarify whether the induction of SCE frequency is dose dependent, high doses cannot be applied in $G_2$-arrested cells since affected cells do not proceed to metaphase. However, experimental evidence has shown that using the chemically induced PCC method, the mutagenic potential of four selected chemicals that induce cell cycle arrest in $G_2$ phase, could be evaluated more accurately. According to Terzoudi et al.[32], the PCC method has been applied for SCEs scoring in $G_2$ phase after exposure to four different chemicals that induce cell cycle arrest in $G_2$ phase. In these experiments, the application of the PCC method has been shown that the mutagenic potential of these chemicals in terms of SCEs may be evaluated even at exposures that arrest cells in $G_2$ phase[31,32]. Moreover, the higher SCE yield per cell scored in $G_2$ PCCs compared to the number of SCEs in metaphase suggests that using the conventional SCE analysis the mutagenic potential of chemicals that temporarily arrest cells in the $G_2$ phase of the cell cycle could have been underestimated[31,32]. Since the scoring of SCEs in human peripheral blood lymphocytes is extensively used as a cytogenetic assay for biomonitoring and genotoxicity testing of potentially mutagenic and carcinogenic chemicals, the combination of PCC with SCEs analysis promises to offer valuable information in genetoxicity studies[4].

## The Uses of PCC in Clinical Cytogenetics and Experimental Therapeutics

The fact that chromosomal analysis by means of the PCC method requires a small amount (0.5 ml or less) of sample (i.e., peripheral blood or bone marrow) makes it especially suitable for *in vivo* and *in vitro* studies, and also in clinical applications for diagnostic purposes. In particular, the ability to visualize the interphase chromosomes of bone marrow and blood cells using PCC by means of mitotic cell fusion with interphase cells has proved useful and accurate in the study of human acute leukemia[91,92,93]. Based on the observation that on growth limitations, normal cells arrest in early $G_1$ phase, whereas transformed cells tend to accumulate in late $G_1$ phase, Hittelman et al. measured the fraction of $G_1$ cells in late $G_1$ phase [proliferative potential index (PPI)] by the use of interphase PCCs in leukemia patients who had achieved complete remission. In a series of studies, they showed that there is a strong correlation between high PPI values and a prediction of early relapse, whereas low PPI values were correlated with continued complete remission[91,92] It has also been shown that the PPI can be used as a predictive indicator for relapse for patients with acute lymphoblastic leukemia (ALL) who have previously relapsed and in myeloid leukemia patients[93].

Recently, calyculin-A-induced PCC has been combined with multicolor FISH [pq-COBRA-FISH (COmbined Binary RAtio labeling-fluorescence i*n situ* hybridization)] for the cytogenetic analysis of cancer cell lines[94]. The use of this technique allows the molecular karyotyping of PCC spreads. In this study, the comparison between PCC spreads and metaphases from mitotic arrest reveals no discrepancies in karyotyping. Interestingly, pq-COBRA-FISH on PCC spreads from fresh colon tumor samples revealed only numerical and no structural abnormalities[94]. Overall, this chemical approach opens new opportunities for the analysis of the chromosomal constitution of $G_1$ and $G_2$ cancer cells and the study of the role of chromosome instability in cancer development[94]. It is generally accepted that the chromosomal instability is associated with several human genetic diseases, such as ataxia telangiectasia (AT), Fanconi’s anemia (FA), and Bloom’s syndrome (BS), as well as with cancer[4,95,96,97]. Therefore, further studies in interphase cancer cells using PCC in combination with other sophisticated techniques may contribute in cancer research.

In the field of prenatal diagnosis, preliminary attempts have been made to combine calyculin-A–induced PCC with GTG banding for fetus examination[82]. However, results so far have shown that the GTG banding resolution of PCC chromosomes is low and further studies are required before the method can be routinely used in cytogenetic prenatal diagnosis.

Furthermore, a major area of experimental therapeutic research centers on the function of anticancer drugs. In this field, the PCC methodology has been applied to study the effect of several anticancer drugs, such as BCNU, CCNU, Cis-acid, VM-26, adriamycin, and neocarzinostatin, whose mode of action depends on cell cycle arrest[26,98]. To determine whether the $G_2$ arrest after exposure to these anticancer drugs was due to a metabolic block or to damage of genetic material, Rao and Rao employed the PCC methodology to visualize the chromosomes of the $G_2$-blocked cells by fusing them with mitotic CHO cells. The PCCs of the treated cells were scored for their position in the cell cycle and the extent of chromosomal damage in the $G_2$ PCC. The results revealed that most of the $G_2$ PCCs of treated cells were extensively damaged, indicating that the $G_2$ accumulation was due to chromosomal damage rather than a metabolic block[26]. In the field of clinical cancer research, the use of PCC methodology in investigating the mode of action of novel or existing anticancer drugs promises to specify the evaluation of new anticancer drugs as well as to improve their clinical utility.

## CONCLUDING REMARKS AND FUTURE PERSPECTIVES

The use of PCC either by means of cell fusion or chemical induction enables the microscopic visualization of interphase chromosomes as well as the analysis of structural and numerical aberrations at the $G_1$ and $G_2$ stages of the cell cycle[19,94]. PCC has been successfully used in problems involving cell cycle analysis[99,100], in the assessment of interphase chromosome malformations resulting from clastogenic treatment with radiation or chemicals[35,98,101,102,103], in pre- and postnatal diagnostic applications, and for diagnostic purposes in cancer cytogenetics and human luekemia[91,104,105]. In particular, the PCC method has proved to be unique in visualizing chromosome damage in cells blocked in $G_2$ phase[98] as well as in old cells incapable of division[106]. It has also been successfully used to determine directly the chromosome repair by allowing a period of time after exposure before fusing the cells[35,102].

The PCC analysis seems to overcome some of the disadvantages of conventional analysis at metaphase level and it can be easily applied since it requires only standard cytogenetic laboratory equipment[32,50], thus it can be performed in most biomonitoring laboratories in order to evaluate more accurately the genotoxic effects of environmental factors exposure even at doses that arrest cells in $G_2$ phase.

The combination of the PCC method with other cytogenetic techniques can be a powerful tool to define and analyze genomic changes as a result of exposure to various environmental agents, in all phases of the cell cycle. Because of the complexity of chromosomal rearrangements, the combination of PCC with other sophisticated techniques seems to elucidate the effect of environmental factor on chromosomes. For example, conventional or chemically induced PCC can be combined with several chromosomal staining techniques (Q banding, G banding, C banding, fluorescent-plus-Giemsa) or FISH[107] for the identification of chromosome aberrations in human and rat lymphocytes, bone marrow cells, and human skin fibroblasts after exposure to ionizing radiation or chemical exposure[58,75,108,109,110,111,112] as well as in interphase tumor cells[113,114].

Theoretically, it is possible to view interphase chromosomes of many, perhaps all, animal species by fusing mitotic cells with interphase cells. Until now, the PCC analysis has been applied for biodosimetry and/or genotoxicity studies in numerous types of cells, such as human, monkey, and rat peripheral blood lymphocytes; CHO cells; rodent spleen cells[35,37,39,51,115]; embryonic mouse cells[70]; human fetal cells from amniotic fluid[82]; HeLa cells[100]; fibroblasts[56,115]; as well as plant cells. To date, the PCC method has been successfully applied in chromosomal examination of human mesodermal cells (i.e., peripheral blood lymphocytes, bone marrow, fibroblasts obtained from by skin biopsies); further studies could also focus on the examination of ectodermal material by means of PCC, in order to gain valuable information especially in the cases of presumable mosaicism[106].

The observation that chromatin spermatozoa can undergo PCC after fusion with somatic cells[106] offers the possibility of the direct analysis of male germ cells for chromosomal aberrations. This application, which has not been used extensively, is of great importance in the spectrum of testing mutagens since the ultimate objective of genetic risk assessments is the quantitative estimation of transmitted genetic damage. Chromosome analysis in spermatozoa has been used, among others, for chromosome aberration analysis after exposure to ionizing radiation[116,117,118], microwave irradiation[119], chemical agents (e.g., triethylenemelamine)[120], alkylating agents (e.g., MMS)[121], and pesticides[122]. Apart from biodosimetry studies, the application of PCC in spermatozoa can also be used to determine the segregation of translocations in meiotic processes, thus providing a useful means to assess risks arising from the induction of translocations in spermatogonial stem cells and the heritable translocations[106].

Until now, a considerable variation of chromosome aberrations observed among different individuals has been linked with cigarette smoking, cancer, radiation exposure (e.g., radiotherapy), and chemotherapy[123,124,125,126]. Moreover, recent perspective studies on human populations have indicated a positive correlation between frequencies of chromosome aberrations in peripheral blood lymphocytes and later onset of cancer[4,127,128].

The PCC methodology can also be applied in interspecies comparisons of cytogenetic response to environmental agents, especially in cases where cells are exposed to environmental factors that induce cell cycle delay such as ionizing radiation, anticancer drugs, and certain chemical agents. Moreover, it is well known that cell cycle arrest is observed not only after chemical or irradiation exposure, but also as a result of viral infection[129]. Examples of viruses that cause cell cycle arrest in the $G_2$/M phase are HIV-1[130,131,132], HTLV, adenovirus, polyomavirus, and SV40[133]. The PCC methodology could be a valuable tool to elucidate the molecular mechanisms underlining host-pathogen interactions that cause $G_2$/M arrest. Furthermore, in the fields of diagnosis, preliminary studies show that PCC can be induced in amniotic fluid cells, thus opening new possibilities for the development of a quick and simple method for prenatal chromosome analysis[82].

Overall, the use of PCC examination has been shown to be a powerful cytokinetic and cytogenetic tool, and it also opens new possibilities to answer fundamental questions in the fields of biodosimetry, toxicogenomics, and clinical diagnosis.

## ACKNOWLEDGEMENT

Grant support: Hellenic Institute for Occupational Health and Safety and contract no FIGH-CT-2002-00218 awarded by the European Commission
